# PScnv: personalized self-normalizing CNV detection with a hierarchical multi-phase framework

**DOI:** 10.1093/bioinformatics/btag099

**Published:** 2026-02-26

**Authors:** Xuwen Wang, Zhili Chang, Wansheng Lv, Akhatov Akmal, Xamidov Munis, Xunbiao Liu, Shenjie Wang, Xiaoyan Zhu, Chong Du, Shuqun Zhang, Jiayin Wang

**Affiliations:** The Comprehensive Breast Care Center, the Second Affiliated Hospital of Xi’an Jiaotong University, No. 157 Xiwu Road, Xi’an 710004, China; School of Computer Science and Technology, Xi’an Jiaotong University, 28 Xianning West Road, Xi’an 710049, China; Shaanxi Engineering Research Center of Medical and Health Big Data, Xi’an Jiaotong University, 28 Xianning West Road, Xi’an 710049, China; School of Computer Science and Technology, Xi’an Jiaotong University, 28 Xianning West Road, Xi’an 710049, China; Shaanxi Engineering Research Center of Medical and Health Big Data, Xi’an Jiaotong University, 28 Xianning West Road, Xi’an 710049, China; Nanjing Geneseeq Technology Inc., Nanjing 211800, China; Nanjing Geneseeq Technology Inc., Nanjing 211800, China; Faculty of Artificial intelligence and digital technologies, Samarkand State University, Samarkand 140104, Uzbekistan; Faculty of Artificial intelligence and digital technologies, Samarkand State University, Samarkand 140104, Uzbekistan; Nanjing Geneseeq Technology Inc., Nanjing 211800, China; School of Computer Science and Technology, Xi’an Jiaotong University, 28 Xianning West Road, Xi’an 710049, China; Shaanxi Engineering Research Center of Medical and Health Big Data, Xi’an Jiaotong University, 28 Xianning West Road, Xi’an 710049, China; School of Computer Science and Technology, Xi’an Jiaotong University, 28 Xianning West Road, Xi’an 710049, China; Shaanxi Engineering Research Center of Medical and Health Big Data, Xi’an Jiaotong University, 28 Xianning West Road, Xi’an 710049, China; The Comprehensive Breast Care Center, the Second Affiliated Hospital of Xi’an Jiaotong University, No. 157 Xiwu Road, Xi’an 710004, China; The Comprehensive Breast Care Center, the Second Affiliated Hospital of Xi’an Jiaotong University, No. 157 Xiwu Road, Xi’an 710004, China; The Comprehensive Breast Care Center, the Second Affiliated Hospital of Xi’an Jiaotong University, No. 157 Xiwu Road, Xi’an 710004, China; School of Computer Science and Technology, Xi’an Jiaotong University, 28 Xianning West Road, Xi’an 710049, China; Shaanxi Engineering Research Center of Medical and Health Big Data, Xi’an Jiaotong University, 28 Xianning West Road, Xi’an 710049, China

## Abstract

**Motivation:**

Accurate detection of copy number variations (CNVs) from targeted panel sequencing remains challenging due to limited genomic coverage and pronounced sample-specific biases. Existing normalization strategies, including baseline-cohort, matched-control, and single-sample approaches, often struggle to balance noise suppression with adaptability, leading to inconsistent performance across heterogeneous samples.

**Results:**

We present PScnv, a personalized self-normalizing framework for robust CNV detection from panel sequencing data. PScnv integrates a pre-built panel-of-normals (PoN) with sample-intrinsic stable chromosomes through ridge-regression normalization to generate individualized log_2_ ratio profiles with reduced systematic variation. CNVs are then identified using a hierarchical multi-phase segmentation pipeline incorporating *z*-score pre-partitioning, kernel-based correction, and circular binary segmentation. In 139 clinical tumor samples with orthogonal FISH validation at MET, ERBB2, and MTAP, PScnv showed improved accuracy and robustness over existing methods that do not require patient-matched normal samples, provided that a pre-built PoN cohort is available.

**Availability:**

Source code is available for academic use at https://github.com/lvws/PScnv.

## 1. Introduction

Copy number variations (CNVs) are structural alterations involving deletion or duplication of DNA segments from ∼1 kilobase (kb) to several megabases (Mb), frequently spanning one or more genes and affecting gene dosage and pathway activity (Zhang *et al.* 2009, Beroukhim *et al.* 2010, MacDonald *et al.* 2014). Pathogenic CNVs contribute to a wide spectrum of human disease, including congenital heart disease (Costain *et al.* 2016), Parkinson’s disease (Landoulsi *et al.* 2025), diabetes (de Jesús Ascencio-Montiel *et al.* 2017), autism (Chen *et al.* 2017), and multiple cancers (Steele *et al.* 2022, Wang *et al.* 2024, Nguyen *et al.* 2025). In oncology, targeted capture panel sequencing has become routine, with clinically validated assays (e.g. FoundationOne, MSK-IMPACT) widely adopted across hospitals and accredited testing laboratories (Frampton *et al.* 2013). Compared with whole-genome and whole-exome sequencing, panel-based assays offer lower cost, deeper coverage, and higher assay specificity (Goodwin *et al.* 2016), enabling broad use in settings such as liquid biopsy and cell-free DNA (cfDNA) testing (Merker *et al.* 2018). These advantages have, in turn, positioned targeted panels as a principal platform for CNV assessment in clinical practice. Among computational strategies for CNV detection, only read depth (RD) based methods are generally applicable to targeted panels (Kumar *et al.* 2023, Quinodoz *et al.* 2024), because the restricted genomic footprint limits split read and paired end evidence (Tan *et al.* 2014, Roca *et al.* 2019, Wang *et al.* 2022). RD-based analysis infers copy number by quantifying reads aligned to targeted intervals; in expectation, coverage scales with underlying copy number (Kumar *et al.* 2023, Quinodoz *et al.* 2024). RD-based analysis estimates copy number from coverage over targeted intervals; however, coverage signals are strongly influenced by capture efficiency, GC and repetitive content, and batch artifacts, complicating normalization and undermining reliable inference. (Kumar *et al.* 2023, Quinodoz *et al.* 2024).

To address these challenges, numerous computational methods have been proposed for CNV detection from panel sequencing data. Broadly, these approaches fall into three categories: (i) baseline-cohort methods, which construct expected depth profiles from collections of normal samples to normalize read counts across targeted regions (Plagnol *et al.* 2012, Boeva *et al.* 2014, Talevich *et al.* 2016); (ii) matched-control designs, which incorporate a patient matched normal sample to suppress sample-specific noise and allelic background (Favero *et al.* 2015, Shen and Seshan, 2016); and (iii) single-sample approaches, which attempt to bypass external references by applying statistical or latent factor normalization, or by leveraging off target reads to approximate genome wide coverage (Kuilman *et al.* 2015, Jiang *et al.* 2018, Laver *et al.* 2022). Although each strategy has demonstrated utility, important limitations remain. First, baseline cohort models reduce random variation but often fail to capture sample-specific biases, limiting accuracy for individual cases (Boeva *et al.* 2014, Talevich *et al.* 2016); Second, matched controls can mitigate sample-specific noise, yet they require additional sequencing that increases cost and may introduce batch related variability; in many workflows, such as germline CNV detection or cfDNA based analyses, a corresponding matched normal is not collected (Martignano *et al.* 2021, Munté *et al.* 2024). Even when available, differences in collection timing, source material, or sequencing runs can introduce further artifacts. Third, single-sample methods, while convenient, often perform less accurately than approaches that incorporate reference cohorts or PoN, since the absence of an external reference makes it difficult to account for systematic biases, an observation reported in multiple benchmarks and best-practice recommendations (Talevich *et al.* 2016, Moreno-Cabrera *et al.* 2020, Munté *et al.* 2024). Moreover, single-sample approaches remain sensitive to residual technical noise, and their reliance on off target coverage or simplified reference models can compromise robustness, particularly for small or heterogeneous panels (Kuilman *et al.* 2015, Jiang *et al.* 2018, Laver *et al.* 2022).

We developed PScnv, a CNV detection framework for targeted panel sequencing that combines personalized self-normalization with hierarchical multi-phase analysis. PScnv uses ridge regression to integrate a PoN baseline cohort with sample-intrinsic stable chromosomes, producing individualized log_2_ ratio profiles that suppress random variation while accommodating sample-specific biases. CNV breakpoints are then refined using a multi-phase segmentation pipeline consisting of *z*-score pre-partitioning, kernel-based signal correction, and circular binary segmentation (CBS). PScnv assigns discrete CNV states and provides gene-level annotation to support downstream interpretation. On 139 clinically profiled tumors with FISH ground truth, PScnv consistently outperformed representative tools.

## 2. Methods

### 2.1.Sample collection and bioinformatics pipeline

#### 2.1.1. Samples from human subjects

We retrospectively reviewed the clinical-genomic database of 139 cancer patients whose tumor samples underwent next-generation sequencing (NGS) and fluorescence in situ hybridization (FISH) at Geneseeq between January 2024 and September 2025. All participants were of Chinese ethnicity. The study was conducted in accordance with the Declaration of Helsinki and was approved by the Medical Ethics Committee of Nanjing Geneseeq Medical Laboratory (NSJB-MEC-2025–11). Written informed consent for research use was obtained from all participants.

To evaluate copy number accuracy, we benchmarked PScnv against FISH as the clinical reference. We analyzed clinical tumor samples with available FISH results for MET, ERBB2, and MTAP, including 86 samples for MET amplification (34 FISH positive and 52 FISH-negative; mainly non-small-cell lung cancer, with a few gastric and vaginal cancers), 27 samples for ERBB2 amplification (14 FISH positive and 13 FISH negative; breast cancer), and 26 samples for MTAP deletion (18 FISH positive and 8 FISH negative; primarily colorectal cancer, glioblastoma, and lung cancer, with a few endometrial and pancreatic cancers). CNV calls were compared with binary FISH results to compute performance metrics, including sensitivity and specificity.

#### 2.1.2. In silico simulated datasets

To systematically evaluate our algorithm across a wide range of controlled conditions, we generated a comprehensive set of in silico datasets using CNV-Sculptor (Chang *et al.* 2025). The simulation produced 50 groups, each containing 10 CNVs of random lengths. For each group, we simulated six tumor purities (0.2, 0.3, 0.4, 0.6, 0.8, and 1.0). For each CNV event, the copy-number state was randomly assigned from eight distinct states (0, 1, 3, 4, 5, 6, 8, and 10). In total, this yielded 3,000 simulated CNV events (10 CNVs per group × 50 groups × 6 purity levels) for robust performance validation.

#### 2.1.3. DNA extraction, library preparation, and targeted sequencing

All experimental procedures, including DNA extraction, library preparation, and targeted sequencing, were performed at Nanjing Geneseeq Technology Inc. (Nanjing, China), a laboratory accredited by the Clinical Laboratory Improvement Amendments (CLIA) and the College of American Pathologists (CAP), following a previously described protocol (Wang *et al.* 2022). For formalin-fixed paraffin-embedded (FFPE) samples, tissues were deparaffinized with xylene and DNA was extracted using the QIAamp DNA FFPE Tissue Kit according to the manufacturer’s instructions. DNA purity and concentration were assessed using a NanoDrop 2000 spectrophotometer and a Qubit 3.0 fluorometer, respectively. Sequencing libraries were prepared from qualified DNA with the KAPA Hyper Prep Kit, followed by hybridization-capture enrichment using a pan-cancer panel targeting 437 cancer-related genes (GeneseeqPrime™, Nanjing Geneseeq Technology Inc., Nanjing, China) with the xGen Lockdown Hybridization and Wash Reagents Kit. Captured libraries were sequenced on the DNBSEQ-T7 platform according to the manufacturer’s protocol.

#### 2.1.4. Bioinformatics analysis

Adapter trimming and low-quality base filtering were performed with fastp (version 0.20.0) (Chen *et al.* 2018). The filtered reads were aligned to the UCSC hg19 (GRCh37) reference genome using the BWA-MEM algorithm (version 0.7.17). PCR duplicates were identified and removed using Picard Tools (Broad Institute).

### 2.2. PScnv workflow

**Figure 1:**
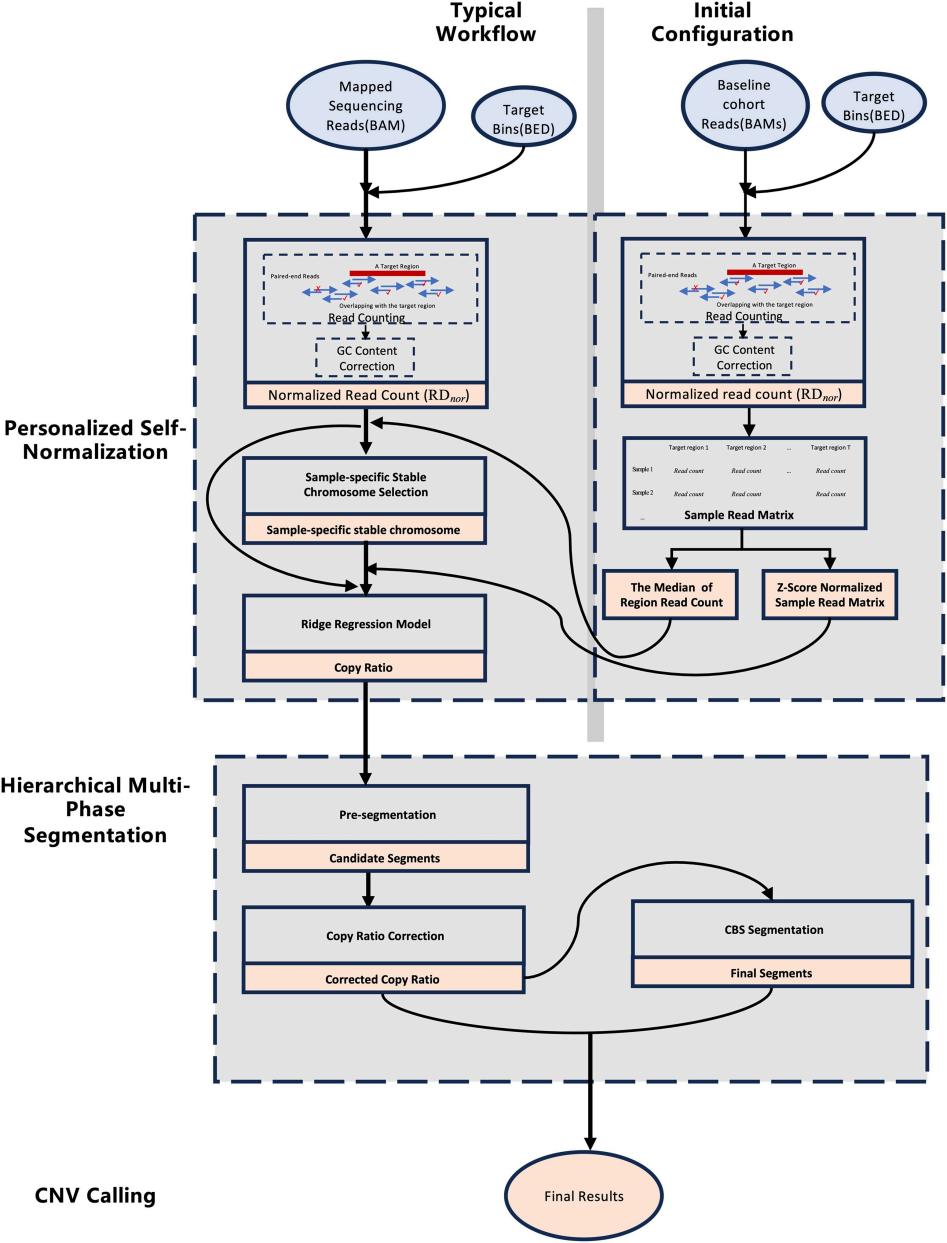
The workflow of PScnv.

PScnv comprises three stages: (i) personalized self-normalization to generate individualized log_2_ ratio profiles; (ii) hierarchical multi-phase segmentation to identify CNV breakpoints; and (iii) CNV calling to assign discrete copy-number states using quality-controlled thresholds. The workflow is shown in Fig. 1.

#### 2.2.1. Personalized self-normalization

##### 2.2.1.1. Read counting

PScnv uses a binary alignment map (BAM) file and a file of target exonic regions (TERs) (i.e. capture target intervals) in BED format. Read depth processing was performed using panel sequencing data with genomic targets defined by the capture BED file. For each target region, read counts were obtained by selecting properly paired end reads in which at least one mate overlapped the region, thereby ensuring robust and comprehensive coverage (Fig. 1, S1 Fig). The raw depth of a region was recorded as *RD_r_*. Depth was then corrected for region length as *RD_l_ = RD_r_/L*, where *L* denotes the region length. Next, the GC content of each target region was computed, and the relationship between *RD_l_* and GC content was modeled using locally weighted regression (LOWESS). GC correction was performed by scaling *RD_l_* as *RD_GC_ = RD_l_/(Fit/Median (Fit))*, where *Fit* represents the LOWESS regression estimate. Finally, sequencing depth was globally normalized across regions by dividing each *RD_GC_* by the median of all regions, yielding the normalized depth *RD_nor_*.

##### 2.2.1.2. Determination of the sample-specific stable chromosome

To derive a robust normalization reference, we identified a Samsam specific stable chromosome by jointly evaluating systematic bias and coverage concordance between the test sample and the baseline cohort. Only autosomes (chr1–22) were considered in this analysis; sex chromosomes (chrX/chrY) were excluded to avoid sex specific copy-number differences and variable capture/coverage patterns. We evaluated the utility of stable-chromosome selection on simulated data by comparing ridge models built on the selected stable chromosome versus all panel targets; the stable-chromosome strategy consistently performs better (S2 Fig). For each test sample, the read depth profile was obtained from properly paired reads overlapping the target regions, following Section 2.2.1. To establish a baseline reference (baseRD), we assembled a cohort of M QC-passed white blood cell (WBC) samples from healthy donors as the PoN baseline cohort (default *M* = 1000, S3 Fig). For each target region *i*, the GC-corrected read depth (*RD_GC_*) across the *M* baseline samples was summarized by its median (Eq. (1)), where $c_{ij}$ denotes the GC-corrected read depth of region *i* in the *j-th* baseline sample. The resulting vector {$c_{i}$} represents a one-dimensional baseline reference depth profile for subsequent comparisons. Two complementary metrics were computed for each chromosome *c* to assess stability: (i) the mean absolute log_2_ difference ${AL}_{c}$ (Eq. (2)), and (ii) the Pearson correlation coefficient *PR_c_* (Eq. (3)), measuring concordance of coverage patterns. Both indices were scaled to [0,1] by min–max normalization across chromosomes (Eq. (4) and Eq. (5)), yielding *NA_c_* and *NP_c_*, where *NA_c_* is the min–max normalized ${AL}_{c}$ and *NP_c_* is the min–max normalized *PR_c_* for chromosome *c*. Each chromosome was thus represented by the point (*NAc, NPc*) in a two-dimensional space. An “ideal” point (0,1) corresponding to zero bias and perfect correlation was defined, and the Euclidean distance to this point was computed as calculated *d* (Eq. (6)). The chromosome with the minimal *d* was designated the stable chromosome for that sample.


(1) $$c_{i}=\mathrm{median}(c_{i1},c_{i2},\ldots ,c_{iM})$$

(2) $${AL}_{c}=|\frac{1}{n}\sum_{i=1}^{n}\;(\log_{2}({\mathrm{testRD}}_{i})-\log_{2}({\mathrm{baseRD}}_{i}))|$$

(3) $${PR}_{c}=\mathrm{Corr}(\mathrm{testRD},\mathrm{baseRD})$$

(4) $${NA}_{c}=\frac{\;{AL}_{c}-\min({AL}_{c})}{\max({AL}_{c})-\min({AL}_{c})}$$

(5) $${NP}_{c}=\frac{{PR}_{c}-\min({PR}_{c})}{\max({PR}_{c})-\min({PR}_{c})}$$

(6) $$d=\sqrt{{\left({NA}_{c}-0\right)}^{2}+{\left({NP}_{c}-1\right)}^{2}}$$

The chromosome with the smallest distance *d* was selected as the stable chromosome for that sample and used as the individualized reference for self-normalization. When *NA_c_*→0, the test and baseline depth profiles exhibit minimal systematic shift in log space, consistent with stable copy number. When *PR_c_*→1, their coverage patterns are highly concordant, indicating limited random drift. By jointly scaling and integrating these indices, the method yields a distance measure that is comparable across chromosomes despite differing depth ranges.

##### 2.2.1.3. Ridge regression model

PScnv performs personalized self-normalization using a ridge regression model that integrates an external baseline cohort with within-sample stability. This approach preserves sample-specific depth patterns while maintaining the noise-suppression benefits of the cohort baseline.

*Data standardization*. Let $X\in R^{N\times M}$ denote the matrix of log2 GC-corrected read depths read depths from the baseline cohort on the stable chromosome, where *N* is the number of target regions and *M* is the number of baseline samples. Each entry *X*_*ij*_ is the GC-corrected read depth for region *i* in baseline sample *j*. For each baseline sample *j*, compute the column mean ${\bar{X}}_{j}$ (Eq. (7)) and standard deviation $s_{j}$ (Eq. (8)), and standardize as ${\tilde{X}}_{ij}$ (Eq. (9)), yielding the normalized baseline feature matrix ${\tilde{X}}_{ij}$. Let $y={\left(y_{1},y_{2},\ldots ,y_{N}\right)}^{⊤}\in R^{N}$ be the vector of log2 GC-corrected read depths for the test sample on the same *N* regions of the stable chromosome, where *y*_*i*_ is the observed log2 GC-corrected read depth for region *i*.


(7) $${\bar{X}}_{j}=\frac{1}{N}\sum_{i=1}^{N}\;X_{ij}$$

(8) $$s_{j}=\sqrt{\frac{1}{N}\sum_{i=1}^{N}\;{\left(X_{ij}-{\bar{X}}_{j}\right)}^{2}}$$

(9) $${\tilde{X}}_{ij}=\frac{X_{ij}-{\bar{X}}_{j}}{s_{j}}$$

*Ridge fitting*. We model the expected copy neutral reference depth of the test sample at region *i* as ${\hat{y}}_{i}$ (Eq. 10), where *w*_*j*_ denotes the contribution of the *j-th* baseline sample, *b* is the intercept, and ${\hat{y}}_{i}$ is the predicted log2 depth for region *i*. The coefficient vector *w* and intercept *b* are estimated by minimizing the ridge-regularized least-squares objective (Eq. 11), where *N* denotes the number of target regions on the sample-specific stable chromosome, ${\tilde{x}}_{i}$ denotes the baseline feature vector for the *i*-th target region across the *M* baseline samples (i.e. the *i*-th row of the standardized design matrix $\tilde{X}$), and let *α *= 1.0 (chosen as a stable default; see S4 Fig.) control the regularization strength. Closed-form estimates for the ridge regression parameters are given by $w^{*}$ (Eq. (12)) and $b^{*}$ (Eq. (13)). These expressions are derived by centering both the response vector and the standardized baseline feature matrix so that the intercept can be handled separately. Specifically, $\bar{y}=1/n\sum_{i=1}^{n}\;y_{i}$ denotes the mean log2 depth of the test sample across the *N* target regions, and $\bar{\tilde{x}}=1/n\sum_{i=1}^{n}\;{\tilde{x}}_{i}$ denotes the mean baseline feature vector.


(10) $${\hat{y}}_{i}=\sum_{j=1}^{M}\;w_{j}{\tilde{X}}_{ij}+b$$

(11) $$\underset{w,b}{\min}\;\frac{1}{N}\sum_{i=1}^{N}\;{\left(y_{i}-(w^{⊤}{\tilde{x}}_{i}+b)\right)}^{2}+\alpha \|w{\|}_{2}^{2}$$

(12) $$w^{*}={\left({\tilde{X}}^{⊤}\tilde{X}+\alpha I\right)}^{-1}{\tilde{X}}^{⊤}y$$

(13) $$b^{*}=\bar{y}-w^{{*}^{⊤}}\bar{\tilde{x}}$$

*Prediction and correction*. The fitted coefficients $\left(w^{*},b^{*}\right)$ were applied to the standardized feature vector ${\tilde{x}}_{i}$ for each panel target region *i* to predict the expected diploid reference value ${\hat{y}}_{i}^{\left(c\right)}$ (Eq. 14). The ridge regression model is fitted using only the target regions on the selected stable chromosome, and the resulting parameters are then applied to targets on all chromosomes. Subtracting the predicted reference from the observed GC-corrected log2 read depth yields the self-normalized log2-ratio $r_{i}^{\left(c\right)}$ (Eq. 15), where *c* indexes the chromosome.


(14) $${\hat{y}}_{i}^{\left(c\right)}={w^{*}}^{⊤}{\tilde{x}}_{i}+b^{*}$$

(15) $$r_{i}^{\left(c\right)}=y_{i}-{\hat{y}}_{i}^{\left(c\right)}$$

By explicitly integrating external baseline information with sample-intrinsic stable regions, this personalized self-normalizing strategy reduces random variation, adapts to sample-specific artifacts that baselines alone cannot address, obviates the need for patient-matched normals by leveraging PoN, and achieves greater robustness than single-sample approaches that lack an external reference.

#### 2.2.2. Hierarchical multi-phase segmentation

PScnv uses a hierarchical multi-phase segmentation strategy to identify CNV breakpoints. It first pre-segments log_2_ ratio profiles to nominate candidate change points, then performs segment level bias correction to refine signals within preliminary intervals. The corrected profiles are subsequently analyzed by CBS to determine final CNV boundaries. This stepwise procedure improves sensitivity while maintaining robustness to technical noise.

*Pre-segmentation*. To generate preliminary CNV intervals, we analyzed the self-normalized log_2_-ratio profiles from Section 2.1.1 using a sliding-window statistic. For a window containing (*n*) observations (*x_1_, x_2_, …, x_n_*) where *n* is the window size (i.e. the number of target regions included in the sliding window) and *x_i_* denotes the self normalized log_2_ ratio of the *i*-th target region, the window mean (*μ*) and standard deviation (*σ*) were computed (Eq. 16). To prevent instability as *σ* to 0, we used an adjusted variance $\sigma^{\prime }=\max(\sigma ,\min\_sd)$, where *min_sd* is a pre-specified minimum standard deviation. Each new observation was then standardized relative to the current window by a *z*-score (*z*) (Eq. 17), which quantifies the deviation of the incoming point from the local distribution defined by *μ* and $\sigma^{\prime }$. Consecutive observations with *z* ≤*z*_*thresh*_ (default 3.0) were same segment, whereas values exceeding the threshold initiated a new breakpoint.


(16) $$\mu =\frac{1}{n}\sum_{i=1}^{n}\;x_{i},\sigma =\sqrt{\frac{1}{n-1}\sum_{i=1}\;{n(x_{i}-\mu )}^{2}}$$

(17) $$z=\frac{\left|x-\mu \right|}{\sigma^{'}}$$

##### 2.2.2.1. Segment-level bias correction

Following pre-segmentation, preliminary CNV intervals may retain residual noise arising from local fluctuations in the log_2_-ratio profile. To attenuate these artifacts and stabilize breakpoint positions, PScnv applies a kernel-based smoothing procedure at the segment level. For each chromosome, the distance between two loci *i* and *j* within a segment is defined as *d_ij_* (Eq. (18)), where *x*_*i*_ and *x*_*j*_ denote the genomic coordinates of the corresponding target regions. A length scale λ is then computed (Eq. 19) to adaptively normalize distances according to segment size. Given λ, we construct a kernel weight matrix *W* (Eq. 20), where *W_ij_* represents the contribution of observation *j* to the smoothed value at locus *i*. Here, *k* denotes the number of target regions within the smoothing neighborhood of region *i*. The fully smoothed log_2_ ratio at locus *i* is obtained as ${\tilde{v}}_{i}$ (Eq. (21)), with $v_{j}$ representing the self-normalized log_2_ ratio in the pre-segmented interval. To avoid over-smoothing, the final corrected value is expressed as a convex combination of the original and smoothed estimates, $v_{i}^{\text{smooth}}$ (Eq. (22)), where α∈[0,1] controls the trade-off between preserving the original signal (α=0) and full smoothing (α=1). This procedure reduces random fluctuations while preserving the underlying CNV structure, ensuring that subsequent CBS-based segmentation operates on denoised and biologically meaningful signals.


(18) $$d_{ij}=|x_{i}-x_{j}|$$

(19) $$\lambda =\frac{\max x_{i}x_{j}-\min x_{i}x_{j}}{4}+1$$

(20) $$W_{ij}=\frac{\;\text{exp}\;\left(-\frac{d_{ij}}{\lambda }\right)}{\sum_{k}\;\;\text{exp}\;\left(-\frac{d_{ik}}{\lambda }\right)}$$

(21) $${\tilde{v}}_{i}=\sum_{j}\;W_{ij}v_{j}$$

(22) $$v_{i}^{\text{smooth}}=\alpha {\tilde{v}}_{i}+(1-\alpha )v_{i}$$

*Final Segmentation with CBS*. Segmentation analysis aims to divide the genome into regions of constant copy number, providing a more interpretable representation than bin-level profiles in which adjacent targets are intrinsically correlated (Yuan *et al.* 2021). In PScnv, segmentation is implemented through a hierarchical multi-phase framework: *z*-score–based pre-segmentation first identifies candidate breakpoints; kernel-based correction attenuates random fluctuations and stabilizes local signal continuity; and the refined profiles are then subjected to CBS (Olshen *et al.* 2004), implemented in the DNAcopy package in R (Venkatraman and Olshen, 2007), with default parameters to determine final breakpoints and partition the genome into segments. By combining pre-segmentation with CBS, PScnv effectively mimics a dynamic sliding-window strategy, which has been shown to improve accurate CNV detection across different size scales, as demonstrated in PEcnv (Wang *et al.* 2022).

#### 2.2.3. CNV calling

Absolute copy number estimation is performed independently of segmentation. The procedures for assigning absolute copy numbers are largely consistent across existing CNV detection tools, and in PScnv we adopt the established calling functions provided by CNVkit (Talevich *et al.* 2016). PScnv reports three CNV states (duplication, deletion, and neutral), and related outputs such as gene-level annotation and interpretation of copy number events are likewise adapted from CNVkit to ensure consistency with widely used standards.

## 3. Results

To assess the performance and generalizability of PScnv, we benchmarked it on simulated datasets and clinical tumor cohorts against representative CNV callers (CODEX2, CNVkit, XHMM, and FACETS). Each tool was executed using the authors’ recommended workflows with default parameters under the corresponding study designs. We evaluated two settings, non-matched controls and matched tumor–normal controls, using standard metrics (sensitivity, specificity, precision, accuracy, and F1-score).

### 3.1. Benchmarking on simulated datasets

#### 3.1.1. Non-matched control design

**Figure 2:**
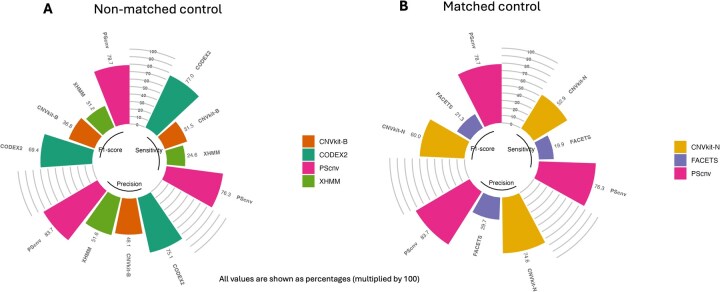
Performance comparison of CNV detection methods on simulated datasets. (A) Non-matched control design: PScnv was compared with CODEX2, CNVkit (unmatched-control mode; CNVkit-B), and XHMM. (B) Matched-control design: PScnv was compared with CNVkit (matched-control mode; CNVkit-N) and FACETS.

To evaluate PScnv without matched controls, we benchmarked it on simulated panel sequencing datasets against representative CNV callers (CODEX2, CNVkit, and XHMM). Because CNVkit can operate in matched and unmatched modes, we used its unmatched configuration to ensure consistency across tools. As shown in Fig. 2A, PScnv achieved the most balanced performance across metrics, with sensitivity = 0.76, precision = 0.84, and F1-score = 0.79. Compared with the next-best method, CODEX2, PScnv increased precision by 9% (0.84 vs. 0.75) and improved F1-score by 10% (0.79 vs. 0.69), while showing a minor decrease in sensitivity of 0.01 (0.76 vs. 0.77). In contrast, CNVkit (unmatched-control mode; CNVkit-B) and XHMM showed substantially lower sensitivity (0.32 and 0.25) and F1-score (0.37 and 0.31), respectively.

#### 3.1.2. Matched-control design

To further evaluate PScnv when matched controls were available, we benchmarked its performance against CNVkit (matched control mode, CNVkit-N) and FACETS using simulated datasets generated under a paired tumor and normal design. As shown in Fig. 2B, PScnv again outperformed the comparator methods across all major evaluation metrics. Specifically, PScnv achieved a sensitivity of 0.76, a precision of 0.84, and an F1-score of 0.79, reflecting marked improvements over CNVkit-N (sensitivity 0.53, precision 0.75, F1-score 0.60) and especially over FACETS (sensitivity 0.20, precision 0.30, F1-score 0.21). Compared with CNVkit-N, PScnv improved precision by approximately 12% and F1-score by nearly 32%, demonstrating its superior capability to maintain accuracy while reducing false positives. Although matched control strategies such as FACETS can effectively suppress sample specific biases through direct tumor normal comparison, their performance is often limited by the quality and batch consistency of control samples. In contrast, PScnv achieves comparable or superior accuracy without the need for strictly paired controls through its personalized self-normalizing framework.

### 3.2. Benchmarking on tumor samples

To comprehensively assess the clinical robustness of PScnv, we compared its performance with representative CNV detection tools: CODEX2, CNVkit-B, XHMM, and FACETS, using 139 tumor samples validated by FISH. The overall benchmarking results are summarized in Table 1. Across all metrics, PScnv achieved the highest and most balanced performance, with sensitivity = 0.86, specificity = 0.88, precision = 0.86, accuracy = 0.87, and F1-score = 0.86. Compared with the best-performing conventional methods, including CNVkit-B (F1-score = 0.77) and CNVkit-N (F1-score = 0.76), PScnv improved F1-score by approximately 12%. While CODEX2 exhibited moderate specificity (0.89), its sensitivity (0.46) was substantially lower, reflecting a strong bias toward false negatives. Conversely, XHMM achieved perfect specificity (1.00) but suffered from extremely poor sensitivity (0.32), indicating overly stringent detection thresholds that fail to capture true CNVs. FACETS, despite leveraging matched control normalization, attained only moderate sensitivity (0.73) and F1-score (0.75). We next assess the detailed performance of each method under different experimental configurations, focusing on the non-matched control design and matched control design.

**Table 1:** Overall benchmarking results on FISH-validated tumor samples.

Tools	TP	FP	TN	FN	Sensitivity	Specificity	Precision	Accuracy	F1-score
CODEX2	30	8	65	36	0.455	0.890	0.789	0.683	0.577
XHMM	21	0	73	45	0.3182	**1.000**	**1.000**	0.676	0.483
CNVkit-B	53	19	54	13	0.803	0.740	0.736	0.770	0.768
CNVkit-N	38	17	40	7	0.844	0.702	0.691	0.765	0.760
FACETS	33	10	47	12	0.733	0.825	0.767	0.784	0.750
**PScnv**	57	9	64	9	**0.864**	0.877	0.864	**0.871**	**0.864**

Overall metrics are micro-averaged over all evaluable tumor–locus instances across ERBB2/MET/MTAP (pooled over gene-specific FISH assessments rather than per patient). TP/FP/TN/FN are defined against FISH ground truth on the pooled instances. The pooled FISH-labeled instances include 66 FISH-positive cases and 73 FISH-negative cases. For paired tumor–normal methods (CNVkit-N/FACETS), the evaluable set is smaller, with 45 FISH-positive and 57 FISH-negative cases, because N/A cases are excluded rather than counted as false negatives.

Overall metrics are micro-averaged over all evaluable tumor–locus instances across ERBB2/MET/MTAP (pooled over gene-specific FISH assessments rather than per patient). TP/FP/TN/FN are defined against FISH ground truth on the pooled instances. The pooled FISH-labeled instances include 66 FISH-positive cases and 73 FISH-negative cases. For paired tumor–normal methods (CNVkit-N/FACETS), the evaluable set is smaller, with 45 FISH-positive and 57 FISH-negative cases, because N/A cases are excluded rather than counted as false negatives.

#### 3.2.1. Non-matched control design

**Figure 3:**
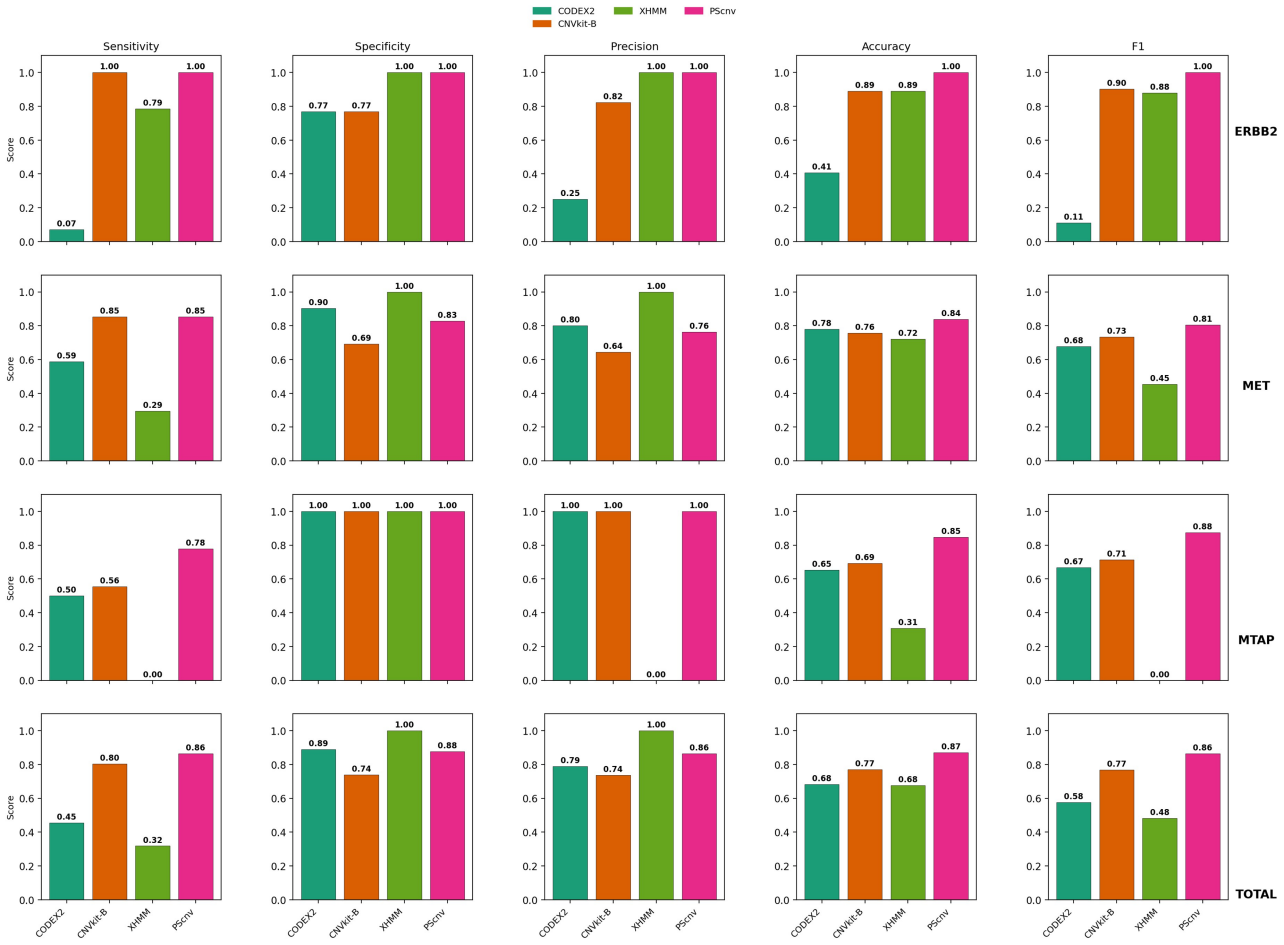
Performance comparison under the non-matched control design on 139 tumors with FISH-validated CNVs at ERBB2, MET, and MTAP, comparing PScnv with CODEX2, CNVkit (unmatched-control mode; CNVkit-B), and XHMM.

Under non-matched control conditions, PScnv consistently outperformed conventional CNV detection tools across **ERBB2**, **MET**, and **MTAP**, suggesting improved robustness in tumors lacking patient-matched normal controls (Fig. 3). For **ERBB2** amplification, PScnv showed perfect agreement with FISH (sensitivity/precision/specificity/accuracy/F1-score, all = 1.00), detecting all amplification events without any false calls. In contrast, CNVkit-B also reached full sensitivity (1.00), but with an 18% reduction in precision (0.82 vs. 1.00), resulting in a lower F1-score (0.90). CODEX2 showed very low sensitivity (0.07), whereas XHMM achieved moderate sensitivity (0.79). For **MET** amplification, PScnv achieved balanced and accurate detection, with sensitivity = 0.85, precision = 0.76, specificity = 0.83, accuracy = 0.84, and F1-score = 0.81. Compared with CODEX2 (F1-score = 0.68) and CNVkit-B (F1-score = 0.73), PScnv achieved a higher F1-score (0.81), representing relative improvements of ∼19% and ∼11%. XHMM, despite perfect precision (1.00), suffered from extremely low sensitivity (0.29), underscoring its over-stringent filtering tendency. For **MTAP** deletion, PScnv yielded the highest sensitivity (0.78) and F1-score (0.88), outperforming CNVkit-B (F1-score = 0.71) and CODEX2 (F1-score = 0.67).

#### 3.2.2. Matched-control design

**Figure 4:**
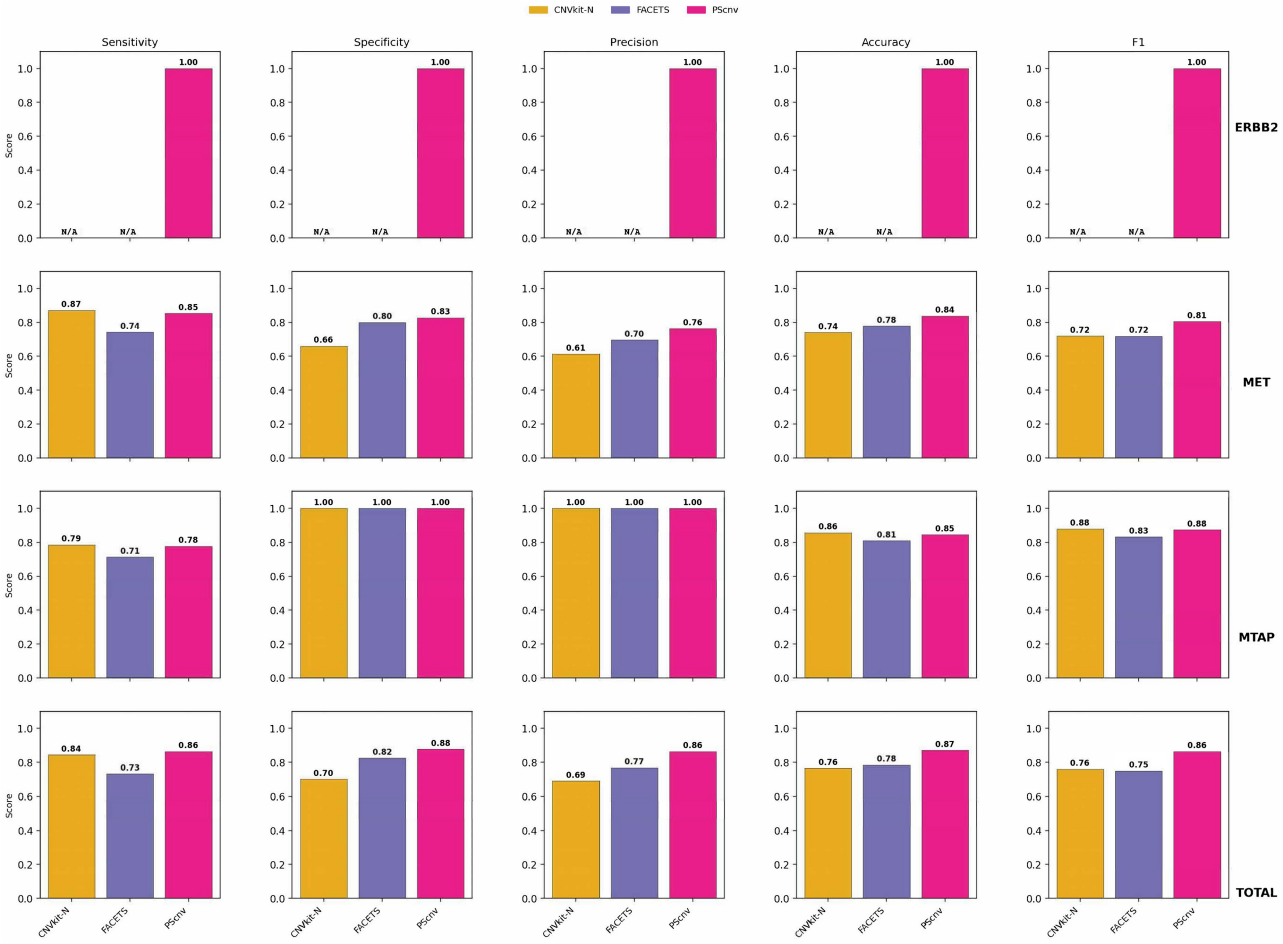
Performance comparison of CNV detection methods under a matched-control design using 139 tumor samples with FISH-validated CNVs at ERBB2, MET, and MTAP. For ERBB2, CNVkit (matched-control mode; CNVkit-N) and FACETS were not evaluable (N/A) because paired tumor–normal samples were unavailable and therefore no valid calls could be generated. “N/A” denotes not evaluable and was not counted as a false negative.

Under the matched-control configuration, PScnv again exhibited consistently superior and stable performance across all genomic loci (Fig. 4). For **ERBB2**, PScnv achieved perfect concordance with FISH (sensitivity = 1.00, specificity = 1.00, precision = 1.00, accuracy = 1.00, F1-score = 1.00). In contrast, CNVkit-N and FACETS could not generate evaluable CNV calls for this locus because matched-control samples were unavailable in the dataset; accordingly, the corresponding evaluation metrics are reported as not evaluable (N/A). For **MET**, PScnv maintained balanced performance, with a sensitivity of 0.85, specificity of 0.83, precision of 0.76, accuracy of 0.84, and F1-score of 0.81. Compared with FACETS and CNVkit-N (both F1-score = 0.72), PScnv increased F1-score by ∼13% and improved precision by ∼9–25% and accuracy by ∼8–14%. For **MTAP**, PScnv achieved F1-score = 0.88 with precision = 1.00, comparable to CNVkit-N (F1-score = 0.88) and higher than FACETS (F1-score = 0.83). While CNVkit-N reached comparable sensitivity (0.79) and F1-score (0.88), FACETS showed a mild decline (F1-score = 0.83).

## 4. Discussion and conclusion

Accurate CNV detection from targeted panel sequencing is limited by two persistent trade-offs: suppressing random noise while adapting to sample-specific biases, and achieving robustness in routine clinical settings while minimizing reliance on matched controls. PScnv addresses these challenges by combining personalized self-normalization with a hierarchical multi-phase analysis. In the normalization stage, PScnv integrates a pre-built PoN baseline cohort with a sample-intrinsic stable chromosome using a ridge regression model. In doing so, it is designed to mitigate random technical variation while adapting to the depth profile of the individual specimen. The subsequent segmentation stage integrates *z*-score based pre-partitioning, kernel-based correction, and CBS-based refinement. These results should be interpreted under practical assumptions: availability of a sufficiently large near-diploid/CNV-negative PoN baseline, adequate TER coverage, and baseline cohort quality with controlled batch/panel effects.

PScnv showed consistently strong and well-balanced performance across both simulated datasets and clinical cohorts. In non-matched settings, using a pre-built PoN baseline cohort, it achieved the highest precision and F1-score while maintaining sensitivity, and this advantage persisted under matched-control designs, outperforming CNVkit (matched mode) and FACETS. These results suggest that cohort-only normalization can underfit or overfit depending on context, whereas sample-aware normalization better preserves true CNV signals while limiting false positives. Clinical validation on 139 tumors with orthogonal FISH further supported robustness, showing perfect concordance for ERBB2 and high accuracy for MET and MTAP under PoN-based non-matched evaluation, and outperforming both matched and unmatched approaches. PScnv reports discrete CNV states using standardized procedures and can be integrated into clinical pipelines. While it does not require patient-matched normal controls, it depends on a reusable PoN baseline built for each panel and workflow, and performance may be influenced by PoN quality and cross-batch or cross-panel differences. When a large PoN is unavailable, users can initialize PScnv with the largest feasible set of CNV-negative normals generated under the same workflow and expand the PoN as additional normals accumulate; we also evaluated performance across PoN sizes (**S3 Fig**). Current limitations include reliance on a high-quality PoN and the lack of cross-panel harmonization. Future work will improve cross-panel portability and automate cohort selection to enhance scalability.

In conclusion, PScnv provides robust CNV calling from targeted panel sequencing via personalized self-normalization and multi-phase segmentation. It achieves high sensitivity with controlled false positives across simulated and clinical datasets, and remains reliable either with matched controls or, when they are unavailable, with a pre-built PoN baseline. PScnv is readily deployable in standard pipelines, addressing major limitations of panel-based CNV detection.

## 5. Key points

- PScnv introduces a personalized self-normalizing strategy that achieves superior accuracy without requiring patient-matched normal controls, by leveraging a pre-built PoN baseline cohort that can be reused across samples processed with the same panel and workflow.
- PScnv introduces a hierarchical multi-phase segmentation scheme that enhances CNV detection accuracy and improves the robustness of CNV profiling in clinical practice.

## Supplementary Material

btag099_Supplementary_Data

## Data Availability

All 139 clinical samples have been deposited in the Genome Sequence Archive (GSA) under accession number HRA014197. The data are publicly accessible at the GSA website (https://ngdc.cncb.ac.cn/gsa/). The Python implementation of PScnv is freely available at GitHub: https://github.com/lvws/PScnv.
