# Supplementary material for: PScnv: personalized self-normalizing CNV detection with a hierarchical multi-phase framework
Source: Bioinformatics. 2026 Feb 26;42(3):btag099. doi: 10.1093/bioinformatics/btag099 (PMC13005925; doi:10.1093/bioinformatics/btag099)
Supplement: btag099_Supplementary_Data [file btag099_supplementary_data.pdf]

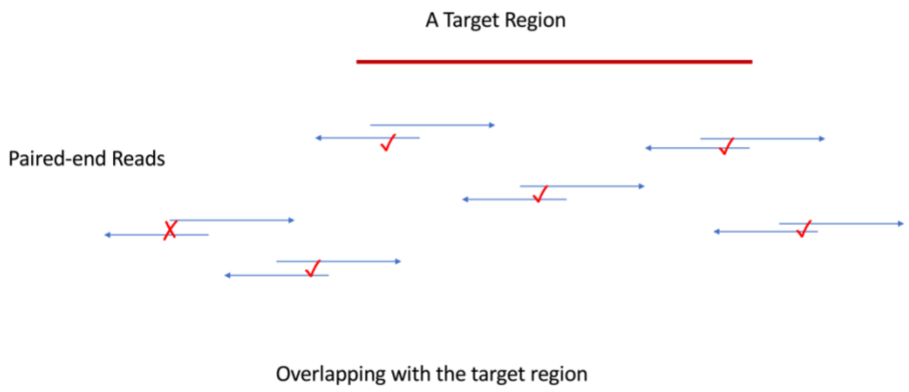

S1 Fig. Schematic illustration of read counting strategy for target regions.

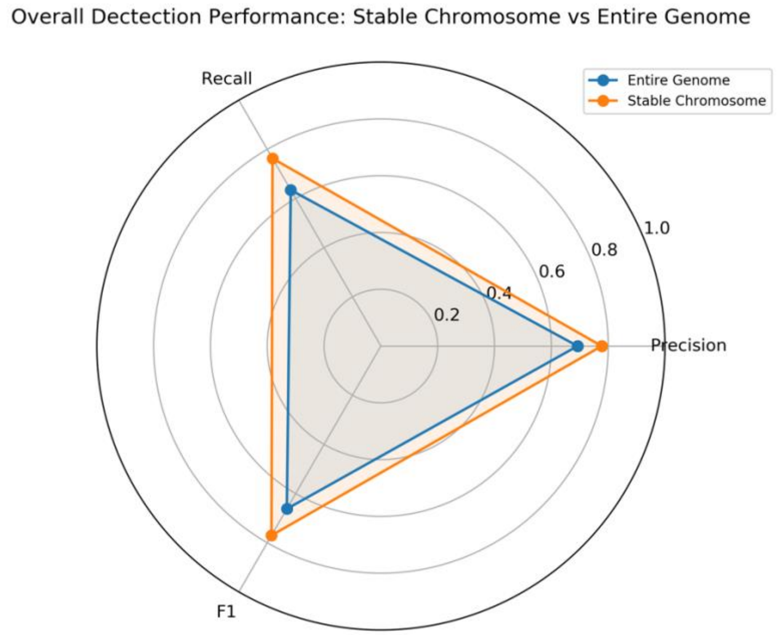

S2 Fig. Ablation analysis of stable-chromosome selection for ridge-regression normalization on simulated data. Radar plot comparing the default PScnv normalization strategy, in which the ridge model is constructed using the sample-specific stable chromosome, with an alternative baseline in which the ridge model is constructed using all panel targets (whole-panel/entire-genome). Performance is summarized using Precision, Recall, and F1-score (range 0–1; higher is better). All other steps (read counting, GC correction, segmentation, and CNV calling thresholds) were kept identical between the two settings. Overall, the stable-chromosome strategy consistently outperforms the whole-panel strategy, improving Precision, Recall, and F1-score on simulated samples.

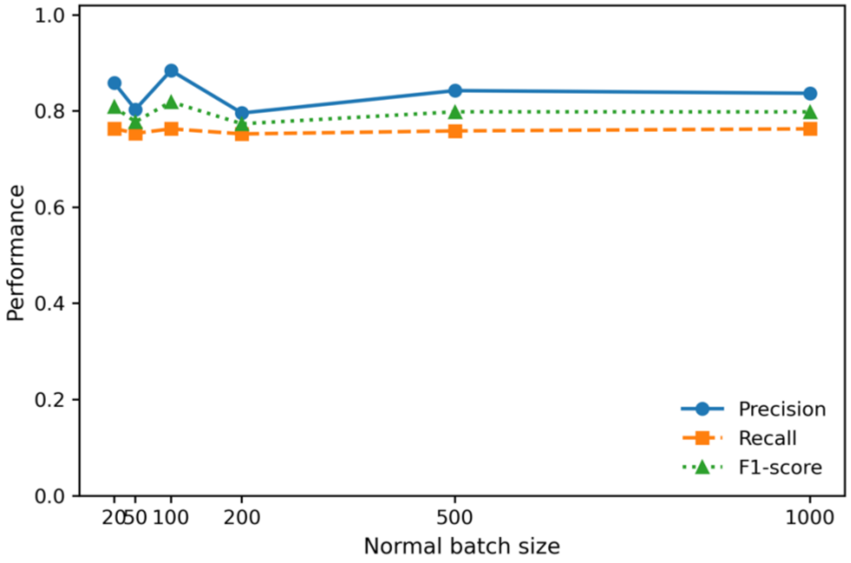

S3 Fig. Effect of baseline cohort size (M) on PScnv performance. Overall performance of PScnv on simulated data was evaluated using different numbers of CNV-negative normal samples to construct the PoN baseline (baseRD). For each M, we report Precision, Recall, and F1-score. All other steps (read counting, GC correction, segmentation, and CNV calling thresholds) were kept identical across M settings.

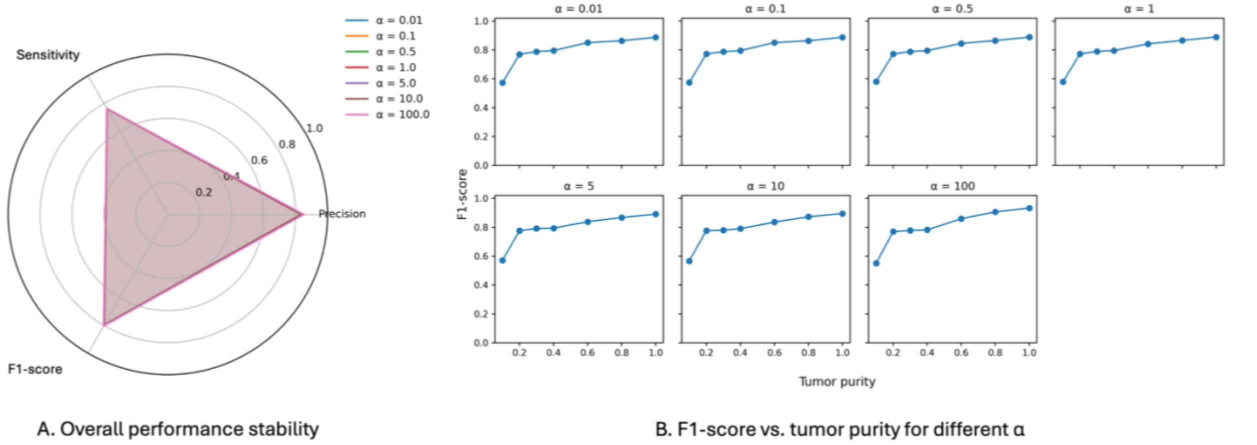

S4 Fig. Robustness of ridge regularization strength (α) and rationale for the default α=1.0 on simulated data. **(A)** Radar summary of overall Precision, Sensitivity, and F1-score for PScnv under a parameter sweep of the ridge penalty α, with α∈{0.01,0.1,0.5,1,5,10,100} (values range from 0 to 1; higher is better). **(B)** F1-score as a function of tumor purity for each α value (one panel per α). Across this range, performance is highly consistent, indicating that PScnv is not strongly sensitive to the exact choice of α. We therefore use α=1.0 as a stable default. All other processing steps and parameters were kept identical across α settings.
